# Essentially exact ground-state calculations by superpositions of nonorthogonal Slater determinants

**DOI:** 10.1186/1556-276X-8-200

**Published:** 2013-05-01

**Authors:** Hidekazu Goto, Masashi Kojo, Akira Sasaki, Kikuji Hirose

**Affiliations:** 1 Department of Precision Science and Technology, Graduate School of Engineering, Osaka University, 2-1 Yamadaoka, Suita, Osaka 565-0871, Japan

**Keywords:** Ground-state calculation, Nonorthogonal Slater determinants, Superposition, Few-electron system, Multiple correction vector

## Abstract

An essentially exact ground-state calculation algorithm for few-electron systems based on superposition of nonorthogonal Slater determinants (SDs) is described, and its convergence properties to ground states are examined. A linear combination of SDs is adopted as many-electron wave functions, and all one-electron wave functions are updated by employing linearly independent multiple correction vectors on the basis of the variational principle. The improvement of the convergence performance to the ground state given by the multi-direction search is shown through comparisons with the conventional steepest descent method. The accuracy and applicability of the proposed scheme are also demonstrated by calculations of the potential energy curves of few-electron molecular systems, compared with the conventional quantum chemistry calculation techniques.

## Background

In recent years, there have been many significant achievements regarding electronic structure calculations in the fields of computational physics and chemistry. However, theoretical and methodological approaches for providing practical descriptions and tractable calculation schemes of the electron–electron correlation energy with continuously controllable accuracy still remain as significant issues [1-15]. Although density functional theory (DFT) supplies a computationally economical and practical method, there are many unexplored problems raised by unreliable results obtained for some systems in which highly accurate electron–electron correlation energy calculations are required, since results by DFT depend significantly on the exchange-correlation energy functional used to perform the calculation [16-18].

The available quantitatively reliable methods require higher computational costs than the DFT method [18]. Although quantum Monte Carlo methods [19-23] can be applied to molecular and crystal systems and show good quantitative reliability where extremely high-accuracy calculations are required, difficulties in calculating forces for optimizing atomic configurations are a considerable disadvantage and inhibit this method from becoming a standard molecular dynamics calculation technique. Configuration interaction (CI), coupled cluster, and Møller-Plesset second-order perturbation methods, each of which use a linear combination of orthogonalized Slater determinants (SDs) as many-electron wave functions, are standard computational techniques in quantum chemistry by which highly accurate results are obtained [24], despite suffering from basis set superposition and basis set incompleteness errors. The full CI calculation can perform an exact electron–electron correlation energy calculation in a space given by an arbitrary basis set. However, it is only applicable for small molecules with modest basis sets since the required number of SDs grows explosively on the order of the factorial of the number of basis.

The required number of SDs in order to determine ground-state energies can be drastically decreased by employing nonorthogonal SDs as a basis set. The resonating Hartree-Fock method proposed by Fukutome utilizes nonorthogonal SDs, and many noteworthy results have been reported [25-30]. Also, Imada and co-workers [31-33] and Kojo and Hirose [34,35] employed nonorthogonal SDs in path integral renormalization group calculations. Goto and co-workers developed the direct energy minimization method using nonorthogonal SDs [36-39] based on the real-space finite-difference formalism [40,41]. In these previous studies, steepest descent directions and acceleration parameters are calculated to update one-electron wave functions on the basis of a variational principle [25-30,36-39]. Although the steepest descent direction guarantees a secure approach to the ground state, a more effective updating process might be performed in a multi-direction search.

In the present study, a calculation algorithm showing an arbitrary set of linearly independent correction vectors is employed to optimize one-electron wave functions with Gaussian basis sets. Since the dimension of the search space depends on the number of linearly independent correction vectors, a sufficient number of correction vectors ensure effective optimization, and the iterative updating of all the one-electron wave functions leads to smooth convergence to the ground states. The primary purpose of this article is to demonstrate the advantage of using multiple correction vectors in searching for the ground state over the conventional steepest descent search in which only one correction vector is used. As a demonstration of the accuracy and applicability of the proposed calculation algorithm, essentially exact potential energy curves of few-electron molecular systems with long interatomic distances are described for cases where the conventional calculation methods of quantum chemistry fail.

The organization of the article is as follows. In the ‘Optimization algorithm’ section, the proposed calculation algorithm for constructing a basis set of nonorthogonal SDs by updating one-electron wave functions with multiple correction vectors is described. The expression of the conventional steepest descent direction with a Gaussian basis set is also given for comparison. The convergence characteristics to the ground states of few-electron systems for calculations using single and multiple correction vectors are illustrated in the ‘Applications to few-electron molecular systems’ section. As demonstrations of the proposed calculation procedure, the convergence properties to the ground states of few-electron atomic and molecular systems are also shown. Finally, a summary of the present study is given in the ‘Conclusions’ section.

## Optimization algorithm

The calculation procedures for constructing a basis set consisting of nonorthogonal SDs for *N-*electron systems using single and multiple correction vectors are described here. An *N-*electron wave function *ψ*(**r**_1_, *σ*_1_, **r**_2_, *σ*_2_,…, **r**_*N*_, *σ*_*N*_) is expressed by a linear combination of nonorthogonal SDs as follows:

(1) $$\begin{array}{l} \psi \left(r_{1},\sigma_{1},r_{2},\sigma_{2},\ldots ,r_{N},\sigma_{N}\right)\\ \;=\sum_{A=1}^{L}C_{A}\Phi^{A}\left(r_{1},\sigma_{1},r_{2},\sigma_{2}\ldots ,r_{N},\sigma_{N}\right). \end{array}$$

Here, **r**_*i *_and σ_*t *_denote the position and spin index of the *i*th electron, respectively. *L* is the number of SDs, and *Φ*^*A*^(**r**_1_, *σ*_1_, **r**_2_, *σ*_2,_…, **r**_*N*_, *σ*_*N*_) is the *A*th SD, given by

(2) $$\begin{array}{l} \Phi^{A}\left(r_{1},\sigma_{1},r_{2},\sigma_{2},\ldots ,r_{N},\sigma_{N}\right)\\ \;=\left|\begin{array}{l} \psi_{1}^{A}\left(r_{1},\sigma_{1}\right)\;\psi_{2}^{A}\left(r_{1},\sigma_{1}\right)\;\ldots \;\psi_{N}^{A}\left(r_{1},\sigma_{1}\right)\\ \psi_{1}^{A}\left(r_{2},\sigma_{2}\right)\;\psi_{2}^{A}\left(r_{2},\sigma_{2}\right)\;\ldots \;\psi_{N}^{A}\left(r_{2},\sigma_{2}\right)\\ \;\vdots \;\vdots \;\ddots \;\vdots \\ \psi_{1}^{A}\left(r_{N},\sigma_{N}\right)\;\psi_{2}^{A}\left(r_{N},\sigma_{N}\right)\;\ldots \;\psi_{N}^{A}\left(r_{N},\sigma_{N}\right) \end{array},\;\right|\\ \;\equiv ||{\vec{\psi }}_{1}^{A}\vec{\psi }{}_{2}^{A}\cdots {\vec{\psi }}_{N}^{A}||\;, \end{array}$$

(3) $$\psi_{i}^{A}\left(r,\sigma \right)=\phi_{i}^{A}\left(r\right)\gamma_{i}\left(\sigma \right),$$

with *ϕ*_*i*_^*A*^(**r**) and *γ*_*i*_(*σ*_*i*_) being nonorthogonal and unnormalized one-electron basis functions and spin orbital functions, respectively. The one-electron wave function *ϕ*_*i*_^*A*^(**r**) is constructed as a linear combination of Gaussian basis functions *x*_*s*_(**r**) [24] as

(4) $$\phi_{i}^{A}\left(r\right)=\sum_{s=1}^{M}D_{i,s}^{A}\chi_{s}\left(r\right).$$

Here, *M* and *D*_*i*,*s*_^*A*^ are the number of basis functions and the *s*th expansion coefficient for the *i*th one-electron wave function *ϕ*_*i*_^*A*^(**r**), respectively.

The steepest direction is implemented in the expression of the total energy functional *E* of the target system on the basis of the variational principle, without the constraints of orthogonality and normalization on the one-electron wave functions. The updating procedure of the *p*th one-electron wave function belongs to the *A*th SD which is represented as

(5) $$D_{p,m}^{A}{}^{\left(\text{new}\right)}=D_{p,m}^{A}{}^{\left(\text{old}\right)}+a_{p}^{A}K_{p,m}^{A},$$

where *a*_*p*_^*A*^ is the acceleration parameter, which is determined by the variational principle with respect to the total energy *E*, i.e., [28]

(6) $$\frac{\mathrm{dE}}{da_{p}^{A}}=0.$$

The component of the steepest descent vector *K*_*p*,*m*_^*A*^ is given by

(7) $$\begin{array}{l} K_{p,m}^{A}=-\frac{\partial E}{\partial D_{p,m}^{A}}\\ \;=-\sum_{B=1}^{L}\left(\frac{\partial \left(\langle H_{0}^{\mathrm{AB}}\rangle +\langle H_{I}^{\mathrm{AB}}\rangle \right)}{\partial D_{p,m}^{A*}}-E\frac{\partial \left|S^{\mathrm{AB}}\right|}{\partial D_{p,m}^{A*}}\right)/\sum_{A=1}^{L}\sum_{B=1}^{L}\left|S^{\mathrm{AB}}\right|, \end{array}$$

where

(8) $$\frac{\partial \left|S^{\mathrm{AB}}\right|}{\partial D_{p,m}^{A}}=\left|S^{\mathrm{AB}}\right|\overset{M}{\underset{j}{\sum }}S_{\mathrm{jp}}^{AB^{-1}}\overset{M}{\underset{n}{\sum }}D_{j,n}^{B}\langle \chi_{m}|\chi_{n}\rangle \delta_{\gamma_{p}\gamma_{j}},$$

(9) $$\begin{array}{l} \frac{\partial \langle H_{0}^{\mathrm{AB}}\rangle }{\partial D_{p,m}^{A}}=\left|S^{\mathrm{AB}}\right|\sum_{i}^{N}\sum_{j}^{N}\sum_{l}^{N}\left(S_{\mathrm{ji}}^{AB^{-1}}S_{lp}^{AB^{-1}}-S_{\mathrm{jp}}^{AB^{-1}}S_{li}^{AB^{-1}}\right)\\ \;\times \sum_{n}^{M}D_{l,n}^{B}\langle \chi_{m}\left|\chi_{n}\right)\rangle \delta_{\gamma_{p}\gamma_{l}}\sum_{s}^{M}\sum_{t}^{M}D_{s,t}^{B}\langle \chi_{s}\left|-\frac{1}{2}\Delta +V\left(r\right)\right|\chi_{t}\rangle \delta_{\gamma_{i}\gamma_{j}}\\ \;+|s^{\mathrm{AB}}|\sum_{j}^{N}S_{\mathrm{jp}}^{AB^{-1}}\sum_{n}^{M}D_{j,n}^{B}\langle \chi_{m}\left|-\frac{1}{2}\Delta +V\left(r\right)\right|\chi_{n}\rangle \delta_{\gamma_{p}\gamma_{j}} \end{array}$$

and

(10) $$\begin{array}{l} \frac{\partial \langle H_{I}^{\mathrm{AB}}\rangle }{\partial D_{p,m}^{A}}=\overset{N}{\underset{l}{\sum }}S_{lp}^{AB^{-1}}\overset{M}{\underset{n}{\sum }}D_{l,n}^{B}\langle \;,\chi_{m}|\chi_{n}\rangle \delta_{\gamma_{p}\gamma_{l}}\langle H_{I}^{\mathrm{AB}}\rangle \\ -\left|S^{\mathrm{AB}}\right|\sum_{I}^{N}\left[\sum_{l}^{N}S_{lI}^{AB^{-1}}\sum_{n}^{M}D_{l,n}^{B}\langle \;\chi_{m}\left|\chi_{n}\right)\rangle \delta_{\gamma_{p}\gamma_{l}}\right]\sum_{J}^{N}\sum_{i}^{N}\sum_{j}^{N}\\ \times \left(S_{\mathrm{Jp}}^{AB^{-1}}S_{\mathrm{ji}}^{AB^{-1}}-S_{\mathrm{jp}}^{AB^{-1}}S_{\mathrm{Ji}}^{AB^{-1}}\right)\times \overset{M}{\underset{s}{\sum }}\overset{M}{\underset{t}{\sum }}\overset{M}{\underset{u}{\sum }}\overset{M}{\underset{v}{\sum }}D_{I,s}^{A*}D_{J,t}^{B}D_{i,u}^{A*}D_{j,v}^{B}\\ \times \langle \chi_{s}\chi_{u}|\frac{1}{\left|r-r'\right|}|\chi_{t}\chi_{v}\rangle \delta_{\gamma_{I}\gamma_{J}}\delta_{\gamma_{i}\gamma_{j}}+\left|S^{\mathrm{AB}}\right|\overset{N}{\underset{J}{\sum }}\overset{N}{\underset{i}{\sum }}\overset{N}{\underset{j}{\sum }}\\ \times \left(S_{\mathrm{Jp}}^{AB^{-1}}S_{\mathrm{ji}}^{AB^{-1}}-S_{\mathrm{jp}}^{AB^{-1}}S_{\mathrm{Ji}}^{AB^{-1}}\right)\times \overset{M}{\underset{t}{\sum }}\overset{M}{\underset{u}{\sum }}\overset{M}{\underset{v}{\sum }}D_{J,t}^{B}D_{i,u}^{A*}D_{j,v}^{B}\\ \times \langle \chi_{m}\chi_{u}|\frac{1}{\left|r-r'\right|}|\chi_{t}\chi_{v}\rangle \delta_{\gamma_{p}\gamma_{J}}\delta_{\gamma_{i}\gamma_{j}}. \end{array}$$

Here, $S_{\mathrm{ji}}^{AB^{-1}}$ denotes the element of the *j*th row and *i*th column of the matrix $S^{AB^{-1}}$. The overlap integral matrix *S*^*AB *^is defined by

(11) $$S^{\mathrm{AB}}=\left[\begin{array}{l} S_{11}^{\mathrm{AB}}\;S_{12}^{\mathrm{AB}}\;\ldots \;S_{1N}^{\mathrm{AB}}\\ S_{21}^{\mathrm{AB}}\;S_{22}^{\mathrm{AB}}\;\ldots \;S_{2N}^{\mathrm{AB}}\\ \;\vdots \;\vdots \;\ddots \;\vdots \\ S_{N1}^{\mathrm{AB}}\;S_{N2}^{\mathrm{AB}}\;\ldots \;S_{\mathrm{NN}}^{\mathrm{AB}} \end{array},\;\right],$$

where the elements *S*_*ij*_^*AB *^are the overlap integrals between the one-electron basis functions, i.e.,

(12) $$S_{\mathrm{ij}}^{\mathrm{AB}}=\int dr\;\phi_{i}^{A\;*}\left(r\right)\;\phi_{j}^{B}\left(r\right).$$

$$\langle {\overset{⌢}{H}}_{0}^{\mathrm{AB}}\rangle$$

and $\langle {\overset{⌢}{H}}_{I}^{\mathrm{AB}}\rangle$ are the matrix elements of the Hamiltonians,

(13) $${\overset{⌢}{H}}_{0}=\sum_{n-1}^{N}\left(-\frac{1}{2}\Delta_{n}+V\left(r_{n}\right)\right)$$

and

(14) $${\overset{⌢}{H}}_{I}=\frac{1}{2}\sum_{n=1}^{N}\sum_{m=1}^{N}\frac{1}{\left|r_{m}-r_{n}\right|},$$

respectively. Here, *V*(**r**) stands for an external potential.

The proposed calculation procedure employs linearly independent multiple correction vectors for updating the one-electron wave function. The *p*th one-electron wave function in the *A*th SD is updated by

(15) $$D_{p,m}^{A}{}^{\left(\mathrm{new}\right)}=D_{p,m}^{A}{}^{\left(\mathrm{old}\right)}+\sum_{\mu =1}^{N_{c}}C_{L+\mu }G_{\mu .m}^{A},$$

where *C*_*j*_(*j* = 1, 2,…, *L* + *N*_*c*_) and *N*_*c*_ are the expansion coefficient and the number of correction vectors, respectively. The components of the correction vectors *G*_*μ*,*m*_^*A*^ determine *N*_*c*_ linearly independent correction functions *ξ*_*μ*_(**r**) which are defined as linear combinations of Gaussian basis functions as

(16) $$\xi_{\mu }\left(r\right)=\sum_{s=1}^{M}G_{\mu ,s}x_{s}\left(r\right).$$

Since the linearly independent correction vectors can be given arbitrarily, randomly chosen values are employed in the present study. A larger number of correction vectors *N*_*c*_ realize a larger volume search space; however, the number of the linearly independent vectors *N*_*c*_ is restricted to the dimension of the space defined by the basis set used.

Thus, we have a linear combination of *L + N*_*c*_ SDs as the new *N*-electron wave function

(17) $$\begin{array}{l} \psi {\left(r_{1},\sigma_{1},r_{2},\sigma_{2},\cdots ,r_{N},\sigma_{N}\right)}^{\left(\mathrm{new}\right)}\\ =\sum_{B=1}^{L}C_{B}\Phi^{B}{\left(r_{1},\sigma_{1},r_{2},\sigma_{2},\cdots ,r_{N},\sigma_{N}\right)}^{\left(\mathrm{new}\right)}\\ =\sum_{B\neq A}^{L}C_{B}||{\vec{\psi }}_{1}^{B}{\vec{\psi }}_{2}^{B}\cdots {\vec{\psi }}_{N}^{B}||\\ +C_{A}||{\vec{\psi }}_{1}^{A}\cdots {\vec{\psi }}_{p-1}^{A}\left({\vec{\psi }}_{p}^{A}+\sum_{\mu =1}^{N_{c}}C_{L+\mu }{\vec{\Xi }}_{\mu }\right){\vec{\psi }}_{p+1}^{A}\cdots {\vec{\psi }}_{N}^{A}||,\\ =\sum_{B=1}^{L}C_{B}||{\vec{\psi }}_{1}^{B}{\vec{\psi }}_{2}^{B}\cdots {\vec{\psi }}_{N}^{B}||\\ +\sum_{\mu =1}^{N_{c}}C_{A}C_{L+\mu }||{\vec{\psi }}_{1}^{A}\cdots {\vec{\psi }}_{p-1}^{A}{\vec{\Xi }}_{\mu }{\vec{\psi }}_{p+1}^{A}\cdots {\vec{\psi }}_{N}^{A}||\equiv \sum_{j=1}^{L+N_{c}}{\tilde{C}}_{j}{\tilde{\Phi }}^{j} \end{array}$$

where

(18) $$\Xi_{\mu }\left(r,\sigma \right)=\xi_{\mu }\left(r\right)\gamma_{i}\left(\sigma \right).$$

**Figure 1:**
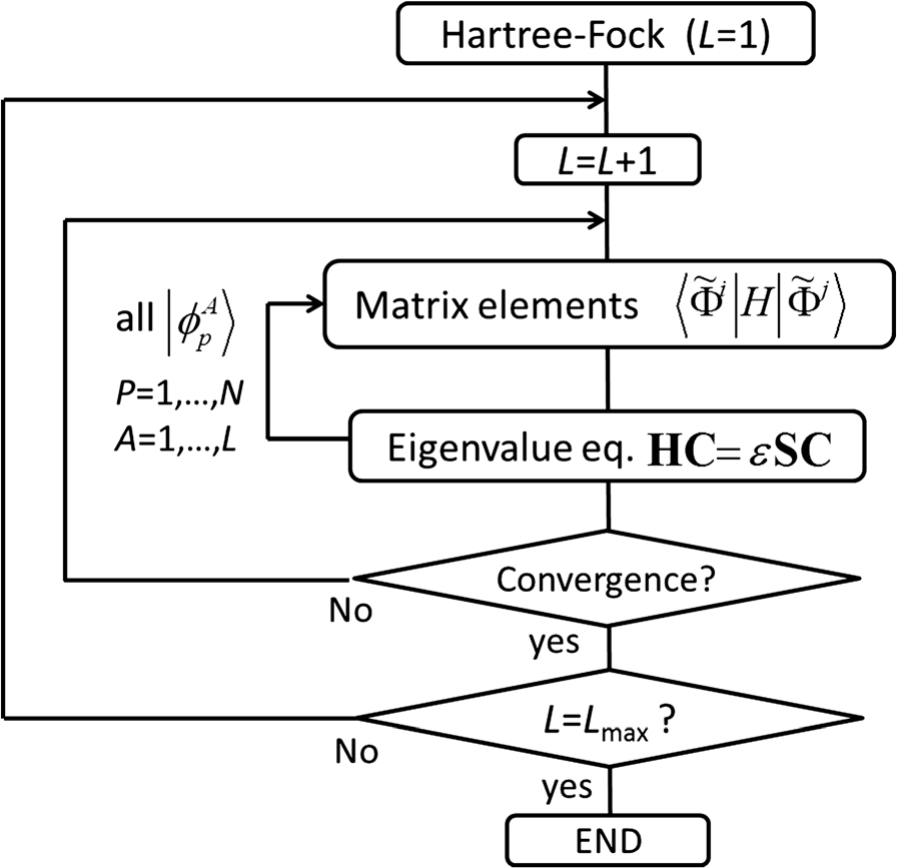
Flow of the present algorithm.

Figure 1 illustrates the flow of the present calculation procedure. Unrestricted Hartree-Fock (UHF) solutions for a target system are used for initial one-electron wave functions. The coefficients ${\tilde{C}}_{j}\left(j=1,2,\ldots ,L+N_{c}\right)$ of Equation 17 are given by solving the generalized eigenvalue equations obtained by employing the variational principle applied to the total energy, and we can have a new *N*-electron wave function as a linear combination of *L* SDs as shown in Equation 17. Iteration of the above updating process for all the one-electron wave functions of all SDs increasing the number of the SDs’ *L* leads to an essentially exact numerical solution of the ground state.

## Applications to few-electron molecular systems

**Figure 2:**
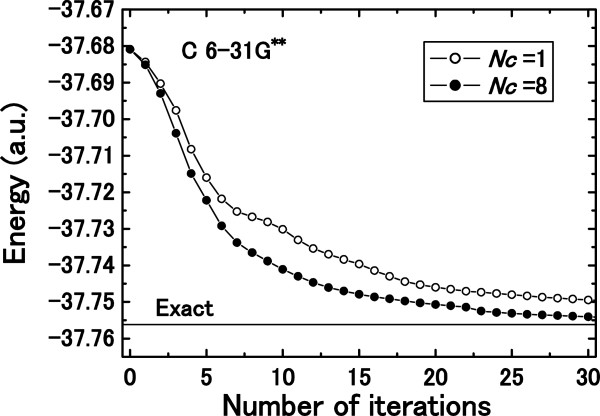
**Effectiveness of multi-direction search on total energy convergence. **Effectiveness of multi-direction search on total energy convergence as a function of the number of iterations for a C atom with the 6-31G** basis set is shown.

Convergence performances for searching for the ground state of a C atom with the 6-31G** basis set are shown in Figure 2. The UHF solutions are adopted as initial states, and the number of employed SDs is 30. The steepest descent direction and acceleration parameter are adopted for the calculation using one correction vector (*N*_*c*_ =1), and seven randomly chosen linearly independent correction vectors are added to the steepest descent correction to create a calculation with eight correction vectors (*N*_*c*_ =8). An indispensable advantage of the multi-direction search over the single steepest descent direction search is clearly demonstrated. Although the steepest descent vector gives the direction with the largest gradient, it does not necessarily point toward the global energy minimum state. On the contrary, a linear combination of multiple correction vectors can be used to obtain the minimum energy state within the given space by adopting the variation principle.

**Figure 3:**
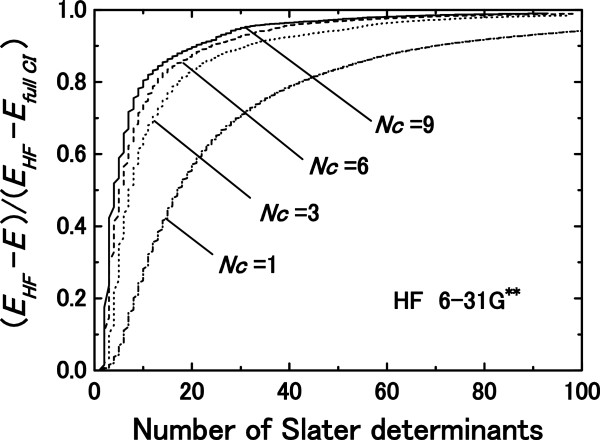
**Convergence performance of the proposed method for the correlation energy.** Convergence performance of the proposed method for the correlation energy of a HF molecule with the 6-31G** basis set as a function of the number of employed SDs is shown.

Figure 3 illustrates the convergence performance of the proposed method for the electron–electron correlation energy of a HF molecule with the 6-31G** basis set as a function of the number of employed SDs. Calculated correlation energies are shown by ratios to exact ones obtained by full CI. The convergence performance to the exact ground state is improved by increasing the number of correction vectors, since the volume of the search space for a one-electron wave function increase with increasing *N*_*c*_. The essentially exact ground-state energy is obtained using less than 100 nonorthogonal SDs with an error of 0.001%, compared with the exact value in which 99.5% of the electron–electron correlation energy is counted. The obtained convergence is so smooth that the accuracy of the total energy is controllable by adjusting the number of employed SDs. On the other hand, the full CI method requires over 10^8^ orthogonal SDs, and thus the reduction in the numbers of SDs is a significant advantage of adopting nonorthogonal SDs. The ground-state energy obtained by the proposed method does not depend on the components of the correction vectors; however, the rate of convergence does depend on the number of employed correction vectors *N*_*c*_.

**Figure 4:**
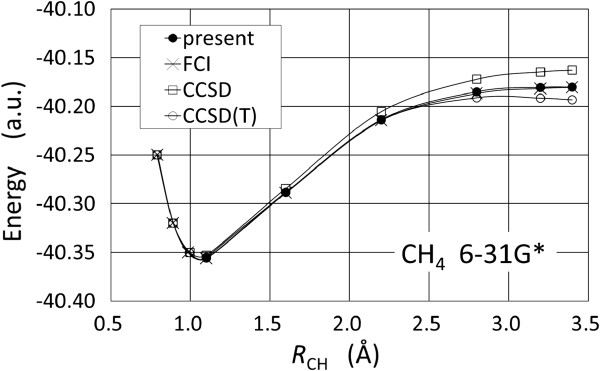
**Potential energy curve of a CH**_**4 **_molecule obtained using the proposed algorithm with 6-31G* basis set.

The potential energy curve calculated when a single H atom is extracted from a CH_4_ molecule as shown in Figure 4. Calculations are performed using the 6-31G* basis set. Although the bond lengths are close to the equilibrium one, the errors in the energies obtained by coupled-cluster theory with singles and doubles (CCSD) plus perturbative triples (CCSD(T)) are a few milliHartree; at longer bond lengths, the accuracy of the results appears to deteriorate [42]. In contrast, the proposed calculation procedure ensures essentially exact ground states at all bond lengths, since no approximations are employed.

**Figure 5:**
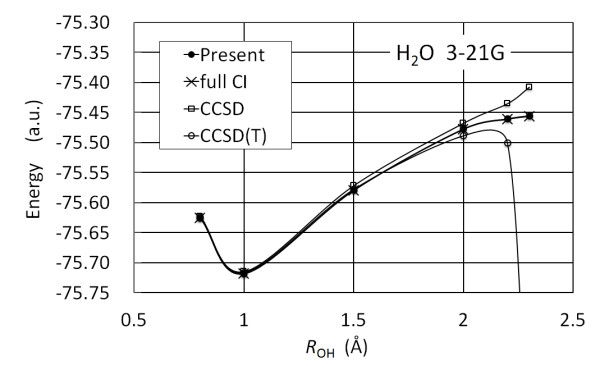
**Potential energy curve of a H**_**2**_**O molecule obtained using the proposed algorithm with 3-21G basis set.**

Figure 5 illustrates the potential energy curve along the symmetric stretching coordinate of a H_2_O molecule in the 3-21G basis set. The angle between the O-H bonds is fixed at 107.6°. These results shown for the proposed calculation method, CCSD and CCSD(T) exhibit the same trends as for a CH_4_ molecule. The results for near the equilibrium bond length demonstrate comparable performance between the four methods, whereas results for long bond lengths indicate only that the proposed method has comparable performance with full CI not producing the same unphysical energy curves as CCSD and CCSD(T) around 2.3 Å [42].

## Conclusions

A reliable and tractable technique for constructing the ground-state wave function by the superposition of nonorthogonal SDs is described. Linear independent multiple correction vectors are employed in order to update one-electron wave functions, and a conventional steepest descent method is also performed as a comparison. The dependence of convergence performance on the number of adopted correction vectors is also illustrated. The electron–electron correlation energy converges rapidly and smoothly to the ground state through the multi-direction search, and an essentially exact ground-state energy is obtained with drastically fewer SDs (less than 100 SDs in the present study) compared with the number required in the full CI method. For the few-electron molecular systems considered in the present study, essentially exact electron–electron correlation energies can be calculated even at long bond lengths for which the standard single-reference CCSD and CCSD(T) show poor results, and the practicality and applicability of the proposed calculation procedure have been clearly demonstrated. In future studies, calculations employing periodic boundary conditions and effective core potentials (ECPs) [43] will be performed. A new procedure to reduce the iteration cost should be found in order to increase the applicability of the proposed algorithm for the calculation of essentially exact ground-state energies of many-electron systems.

## Competing interests

The authors declare that they have no competing interests.

## Authors’ contributions

HG conceived, planned this study, carried out the coding of the computation program, and drafted the manuscript. MK and KH participated in the discussions on the basic theory of the present method. AS performed tunings of the code and made all of calculations. All authors read and approved the final manuscript.
